# Supplementary material for: Tumorigenicity decrease in Bcl-xL deficient MDCK cells ensuring the safety for influenza vaccine production
Source: PLoS One. 2024 Dec 16;19(12):e0311069. doi: 10.1371/journal.pone.0311069 (PMC11649150; doi:10.1371/journal.pone.0311069)
Supplement: S1 File — (ZIP) [file pone.0311069.s002.zip › S2_File/3_KEGG_enrichment/scatter/B_vs_A.down_KEGG_enrichment_scatter.pdf]

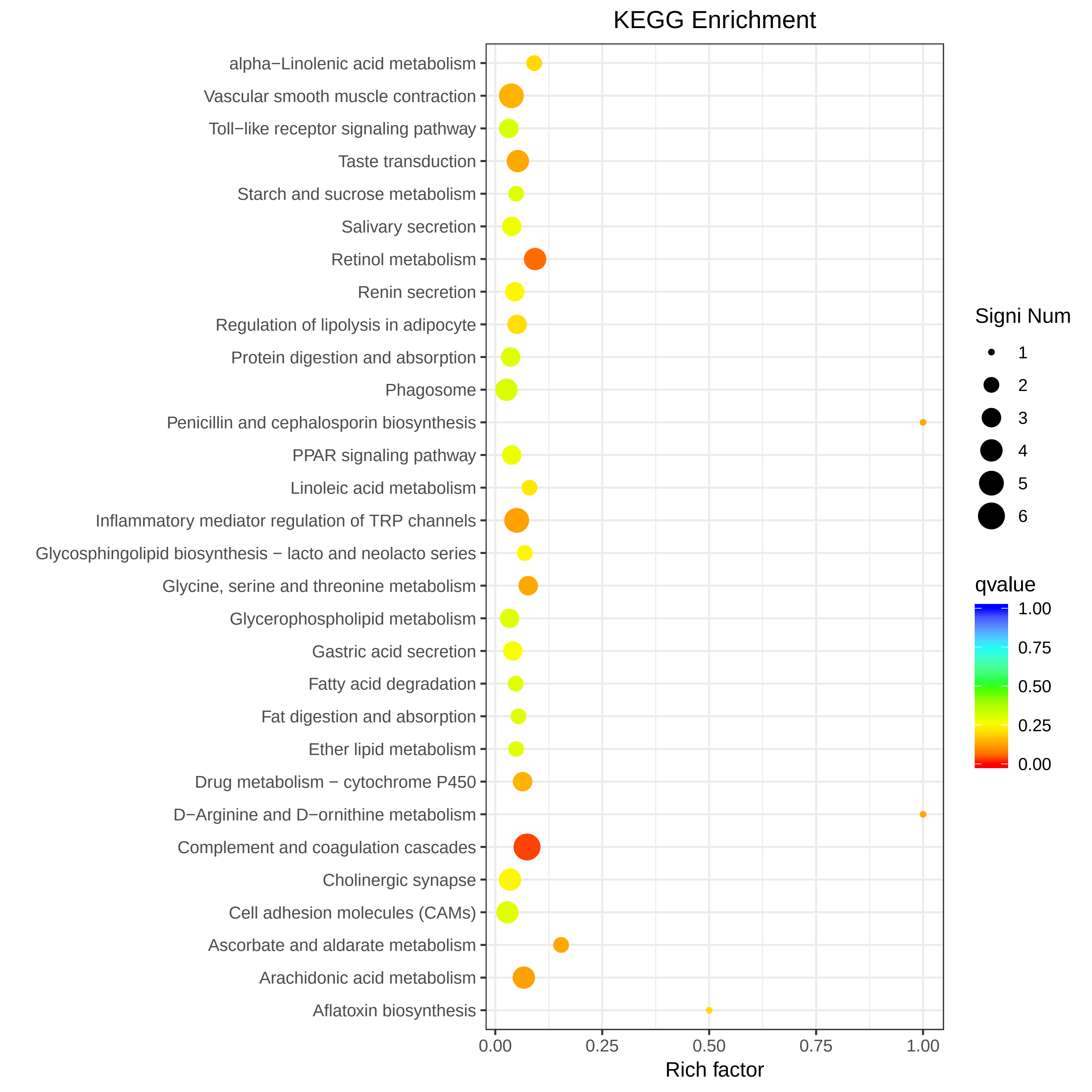
KEGG Enrichment
alpha−Linolenic acid metabolism
Vascular smooth muscle contraction
Toll−like receptor signaling pathway
Taste transduction
Starch and sucrose metabolism
Salivary secretion
Retinol metabolism
Renin secretion
Regulation of lipolysis in adipocyte
Protein digestion and absorption
Phagosome
Penicillin and cephalosporin biosynthesis
PPAR signaling pathway
Linoleic acid metabolism
Inflammatory mediator regulation of TRP channels
Glycosphingolipid biosynthesis − lacto and neolacto series
Glycine, serine and threonine metabolism
Glycerophospholipid metabolism
Gastric acid secretion
Fatty acid degradation
Fat digestion and absorption
Ether lipid metabolism
Drug metabolism − cytochrome P450
D−Arginine and D−ornithine metabolism
Complement and coagulation cascades
Cholinergic synapse
Cell adhesion molecules (CAMs)
Ascorbate and aldarate metabolism
Arachidonic acid metabolism
Aflatoxin biosynthesis
0.00
0.25
0.50
0.75
1.00
Rich factor
Signi Num
1
2
3
4
5
6
qvalue
1.00
0.75
0.50
0.25
0.00
